# Asexuality Development among Middle Aged and Older Men

**DOI:** 10.1371/journal.pone.0092794

**Published:** 2014-03-25

**Authors:** Yan-Ping Huang, Bin Chen, Ping Ping, Hong-Xiang Wang, Kai Hu, Hao Yang, Tao Zhang, Tan Feng, Yan Jin, Yin-Fa Han, Yi-Xin Wang, Yi-Ran Huang

**Affiliations:** Department of Urology, Renji Hospital, School of Medicine, Shanghai Jiao Tong University, Shanghai Institute of Andrology, Shanghai, China; Kaohsiung Chang Gung Memorial Hospital, Taiwan

## Abstract

**Objectives:**

To assess erectile function in middle-aged and older men with asexuality status and further analyze their specific reasons for this condition.

**Subjects and Methods:**

Men who had regular sexual intercourse attempts (sex frequency≥1 time per month) were classified into mild erectile dysfunction (ED), moderate to severe ED and non-ED according to International Index of Erectile Function-5, and men having no sexual intercourse attempts for at least 6 months were defined as having an asexuality status. The risk factors associated with ED were collected in a sample of 1,531 Chinese men aged 40 to 80 years, and the self-report reasons for asexuality were recorded in asexual cohort individually. Comparative analyses and multivariate regression models were conducted among these groups.

**Results:**

The prevalence rates of ED and asexuality status were 49.9% and 37.2%. The asexuality status group had higher risk factors than the moderate to severe ED group in terms of old age (age≥65, adjusted odds ratio (OR) 17.69 versus (Vs.) 7.19), diabetes (crude OR: 2.40 Vs. 2.36) and hypertension (crude OR: 1.78 Vs. 1.72). The specific reasons for the asexuality status were “erectile difficulty” (52.9%), “do not care about sexuality” (53.5%)”, “no longer necessary to have sexuality at this age” (47.7%), “severe stress” (44.4%), “severe fatigue” (26.3%) and “masturbation” (26.9%).

**Conclusions:**

Men with an asexual status suffer from higher risk factors for ED than men with moderate to severe ED. The majority of this asexual status could be attributed to a full ED, although the reasons for this transient asexuality also involved sexual attitudes and interests, sexual partners and masturbation.

## Introduction

With the development of society and the process of aging, medical attention and services relating to sexual function are increasing, and the middle-aged and older adults are the most common target population in many studies for surveying and treating sexual problems. Erectile dysfunction (ED) is the most common sexual problem discussed by a growing mass of studies all over the world, yet there is limited information for the asexuality status which may differ from ED in psychological and physical conditions. As opposed to the permanent asexuality condition afflicting around 2–3% of men and not defined as yet as a disorder [1], [2], an asexuality status may be defined as a temporary or irreversible stage in middle aged and older men who had sexual prior sexual experience but are now in a stage of disinterest towards heterosexual intercourse. Often, health-care professionals consider that an asexuality status is related to psychogenic factors, religion, sexual partners and even homosexuality, and they fail to assess these asexual men, defined as having no heterosexual intercourse for a long time, by applying the International Index of Erectile Function (IIEF) to determine ED. As no standard recommendations for evaluating asexuality status, how to verify the true erectile function and explore the specific reasons for asexuality in this cohort are of particular importance. But to date, no comprehensive, representative and population-based data are available to help physicians understand the status of asexuality. Shanghai took a lead in ageing process and became the first area with an old population structure in China. The number of people over 65 will reach a peak of four million in the year 2025, and then occupy 29% of the total population [3]. Thus the ageing population in Shanghai could be considered to be nationally representative, and the male population could be the optimal sample for studying sexual dysfunction. The aim of this well-designed large population-based study was to verify the erectile function in middle-aged and older men with an asexual status by comparing the asexuality status with ED and non-ED as defined clinically, and in terms of the socio-demographic, clinical and lifestyle characteristics and further analyzing the specific reasons for asexuality.

## Materials and Methods

### Study Population

This study investigated sexuality and health status in middle aged and older men from 40 to 80 years of age. Twenty-two communities were stratified as urban central area, urban outer area and urban fringe area by epidemiologists. Seven communities were confirmed for investigation by a stratified random sampling method. The participants from randomly selected communities were included by posters. During the investigation phase (from 2008 to 2011), men who had self-care ability and resided in the city for more than one year were eligible for interview. The subjects who had congenital developmental disorders and/or congenital deformity, serious diseases (i.e. severe cardiac disease and/or psychiatric disorders, significant renal and/or hepatic dysfunction) and homosexual or bisexual orientation, were excluded in the screening procedure of eligibility. All the disorders were confirmed by self-report, medical record review and interview. Of 1,720 eligible respondents, 1,591 completed the baseline in-home protocol. Of the original 1,591 respondents to the baseline survey, 60 were excluded as the conflicting or incomplete data, which left 1,531 men eligible for the statistics.

### Measures Used

The field protocol was developed according to the model of Massachusetts Male Aging Study [4]. Briefly, a trained field technician/phlebotomist visited each subject in Community Service Center or his home according to standard research protocols developed for large scale fieldwork [5], collected demographic data, administered a general health questionnaire and sexual status assessment instruments, and obtained fasting blood samples. This study received institutional review board approval (Renji Hospital, Shanghai. No. RJLS2008175), and written informed consent was given by all study participants. All collected data were uploaded into a database established by using the ACCESS system plus functional module, which can be found in both Science and Technology Commission of Shanghai and Shanghai institute of Andrology.

Three blood pressure measurements were obtained. Body mass index (BMI) was calculated as measured weight in kilograms divided by measured height in meters squared and categorized using the World Health Organization (WHO) classifications [6]: overweight (≥25 kg/m^2^) or not (<25 kg/m^2^). Waist circumference (WC) measurements were used as a measure of central adiposity, and classified into two categories: obesity (≥90 cm) or not (<90 cm)) [7].

With regard to the assessment of sexual status, a self-administered questionnaire on sexual activity was given to each subject for completion in private. In the baseline sexual status questionnaire men classified themselves into two levels: no sexual intercourse or having sexual intercourse in the past 6 months. A 5-item form of the International Index of Erectile Function (IIEF-5) was privately provided to subjects with a frequency of sexual intercourse ≥1 time per month in the past 6 months, and they were categorized into three levels: non-ED (IIEF-5≥22), mild ED (21≥IIEF-5≥12) and moderate to severe ED (11≥IIEF-5≥5). Respondents with no sexual intercourse attempts for at least 6 months were defined as having an asexuality status. Information related to the asexuality status, including heterosexual partner (“single, widowed, divorced or separated” and “poor sexual relationship”), sexual interests (“do not care about sexuality”), erectile problems (“erectile difficulty”), sexual attitudes (“no longer necessary to have sexuality at this age”), social and life stress (“severe stress”, “severe fatigue” and “low life satisfaction”) and masturbation (“normal masturbatory erection” and “weak masturbatory erection”), were individually collected in the population. Eventually, the specify reasons for asexuality status were distilled from the collected information of interviews.

Data from the baseline interview were used to assess the lifestyle factors of interest. Participants were asked about regular exercise in the past 5 years (“regular” was defined as at least once per week, for more than 3 months continuously) [8]. Subjects’ customary alcohol intake was estimated by self-report using the formula of Khavari and Farber [9]. Exposure to cigarette smoke was ascertained through self-report, and current smokers were defined as if they were smoking at the time of the survey and had smoked more than 100 cigarettes in their lifetime [10]. Drinking tea intake was assessed via a frequency questionnaire for over the past 5 years and categorized into tertiles (“regular” was defined as at least once a day, for more than 1 year continuously).

In order to confirm these self-reported chronic disease outcomes, we used a variety of methods including medical record review, pathology report review, telephone interview, or supplementary questionnaires. Hypertension at baseline was indicated if one or more of the following conditions were met: 1) the subject reported use of antihypertensive medication; 2) the subject’s systolic blood pressure≥140 mmHg or diastolic blood pressure≥90 mmHg [11]. Dyslipidemia was defined as serum total cholesterol≥5.72 mmol/L; and/or triglycerides≥1.70 mmol/L; and/or low density lipoprotein cholesterol≥3.64 mmol/L; and/or use of cholesterol-lowering medication. Diabetes was defined as fasting blood glucose≥7.0 mmol/L and/or use of anti-diabetes medication. Using the National Institutes of Health Chronic Prostatitis Symptom Index(NIH-CPSI), prostatitis-like symptom (PLS) was defined as having lower urinary tract symptoms (LUTS), or/and perineal and/or ejaculatory pain or discomfort [12]. The International prostatic symptom score (IPSS), digital rectal examination (DRE), medical record of ultrasound and receiving anti-androgen medications were used to identify the accuracy of self-reported benign prostatic hyperplasia (BPH).

All study personnel successfully completed a training program that oriented them to both the aims of the study and the specific tools and methodologies used. One tube of fasting blood sample was taken for serum glucose (measured by use of a modified hexokinase enzymatic method) and lipid assays (analyzed enzymatically by use of commercially available reagents) [13]. Two additional tubes of non-fasting blood samples were drawn for hormone assays [14], [15], [16] and total prostate specific antigen (TPSA) [17], respectively. All the blood tests were conducted in clinical laboratory centre (Renji Hospital, Shanghai, China).

### Statistical Analysis

All participants were categorized into four age groups according to the age distribution of the investigated population (40–51, 52–59, 60–64, and 65–80). Sexual status was categorized into four groups: non-ED, mild ED, moderate to severe ED and asexuality status. One-Way ANOVA (data met normal distribution), Kruskal-Wallis (data met non-normal distribution) and Chi-square tests (ranked data) were used to compare among four groups on all related characteristics, and Bonferroni correction was used to counteract the bias of multiple comparisons. Finally, multivariate regression models investigated whether a priori determined general characteristics, clinical and lifestyle characteristics were associated with sexual status. Continuous variables were provided as mean±standard deviation (SD) or median (minimal-maximum). Statistical *P*<0.05 was considered indicative of clinical meaningful differences between groups. All statistical analysis was performed using SPSS13.0 (SPSS Inc., Chicago, Illinois, USA).

## Results

**Figure 1:**
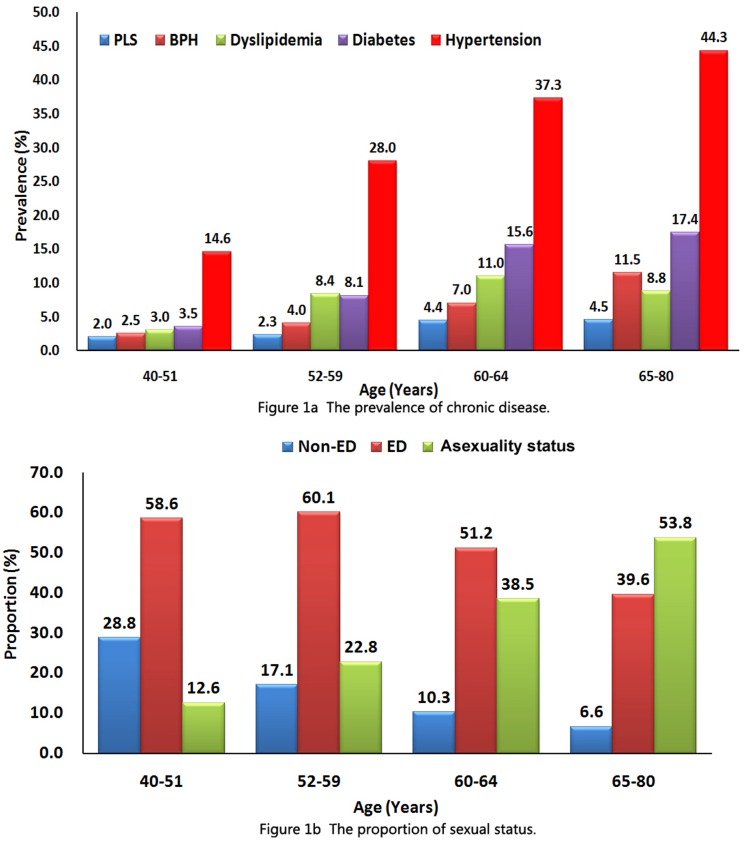
The distribution of chronic disease and sexual status among age groups.

**Figure 2:**
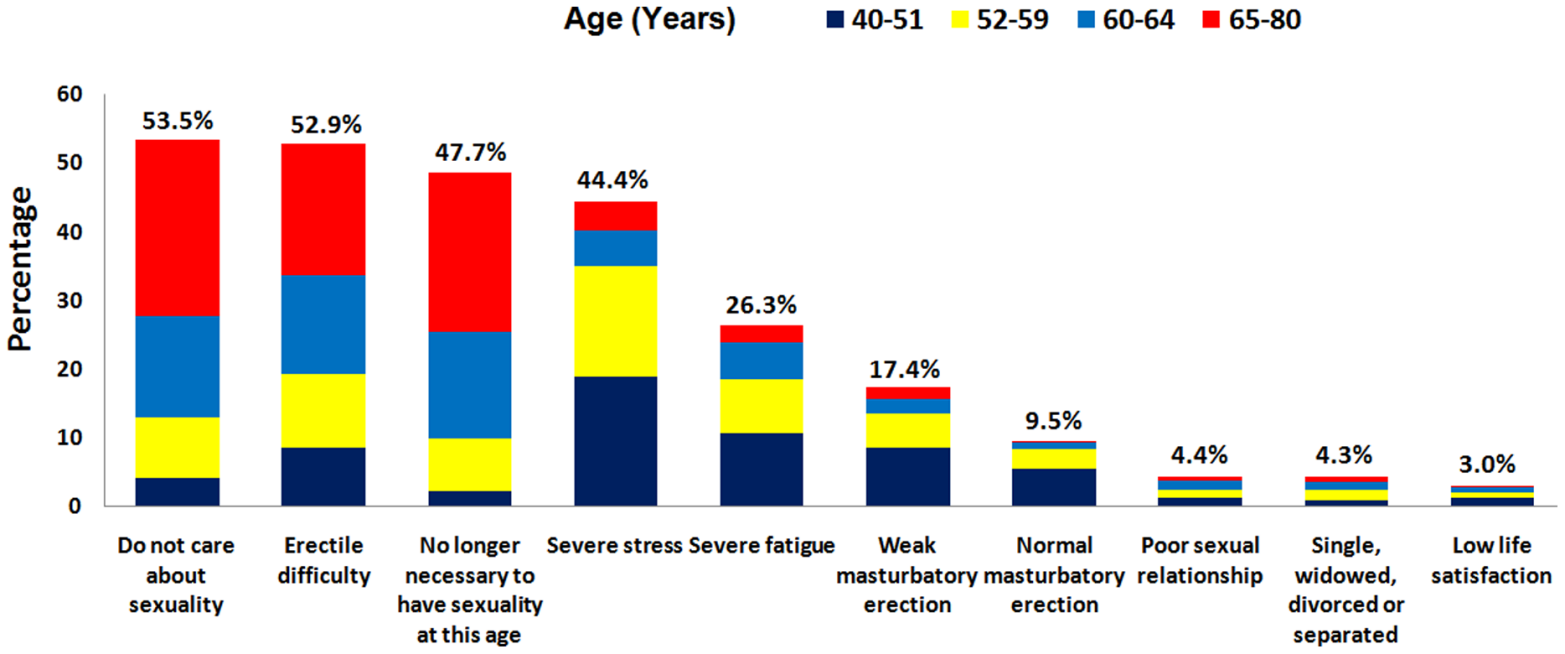
The specific reasons for asexuality in the population without sexual intercourse.

Of 1,720 eligible respondents, we received responses from 1,591 subjects (92.5 percent) and screened samples from 1,531 subjects (89.0 percent). The proportions of different age bracket respondents were 12.9% (40–51), 22.6% (52–59), 28.0% (60–64) and 36.4% (65–80), respectively. The total prevalence of ED and asexuality status were 49.9% (765/1,531) and 37.2% (569/1,531), respectively. The distribution of chronic disease and sexual status among age groups is shown on Figure 1. The specific reasons for asexuality status, including the main reasons “do not care about sexuality” (53.5%), “erectile difficulty” (52.9%), “no longer necessary to have sexuality at this age” (47.7%), “severe stress” (44.4%) and “masturbatory erection” (26.9%), were summarized in Figure 2.

Table 1 summarized the differences of risk factors associated with ED among four groups. The asexuality status population had older age, higher systolic blood pressure, higher FBG, serum creatinine and TPSA level, and lower LH level; and presented higher prevalence of diabetes and hypertension.

**Table 1:** Demographic and clinical characteristics of the participating men according to IIEF-5 score.

	Non-ED (N = 197)	Mild ED (N = 642)	Mo-Se ED(N = 123)	Asexuality(N = 569)	*P*
**Normal data** a	**Mean ±SD**				
** Age**	56.84±8.92	59.77±8.63*^†^	63.06±8.54*	65.70±8.20*^†^	<0.001
** BMI**	24.17±3.54	24.34±3.48	24.50±3.12	24.17±3.66	0.705
** WC**	83.03±6.95	82.82±8.81	84.41±6.49	82.44±7.22^†^	0.091
** Systolic BP**	128.36±16.04	129.58±15.04	128.63±15.50	132.16±17.72*	0.005
** Diastolic BP**	82.25±8.97	82.39±8.87	81.69±9.52	81.76±9.47	0.625
** HR**	74.14±4.58	73.76±5.65	74.09±5.50	74.08±5.59	0.703
** IIEF-5**	23.22±0.96	17.07±2.62*	8.13±2.22*	–	<0.001
** FBG**	5.51±1.71	5.53±1.58	5.59±1.52*	5.95±2.34	0.070
** Creatinine**	74.73±10.49	75.32±13.02	75.19±10.35	76.97±17.01	0.114
** TT**	4.52±2.97	4.49±2.37	4.45±2.15	4.19±2.19	0.629
**Non-normal data** b	**Median (min-max)**				
** TPSA**	0.67 (0.09–13.20)	0.74 (0.09–15.60)	0.82 (0.09–24.00)*	1.04 (0.09–48.00)*^†^	<0.001
** FSH**	5.43 (1.04–60.70)	6.52 (0.43–69.01)	4.65 (0.52–44.70)*	6.80 (0.46–60.70)^†^	<0.001
** LH**	4.19 (0.30–40.70)	3.79 (0.20–30.20)	3.63 (0.79–20.90)	3.13 (0.36–21.90)^†^	0.022
** PRL**	6.88 (0.48–51.27)	7.62 (0.48–252.30)	7.35 (0.83–34.84)	7.78 (0.54–120.20)	0.555
** E_2_**	12.00 (12.00–139.00)	12.00 (0.69–122.00)	12.00 (12.00–104.00)	12.00 (12.00–215.00)	0.813
** T/E_2_**	0.25 (0.03–1.17)	0.26 (0.01–4.74)	0.26 (0.02–0.77)	0.22 (0.01–2.68)	0.359
** ALT**	18.00 (5.00–178.00)	18.00 (3.00–132.00)	18.00 (4.00–86.00)	19.00 (4.00–196.00)	0.913
** TG**	1.50 (0.48–19.23)	1.45 (0.45–15.00)	1.52 (0.43–6.48)	1.52 (0.41–12.54)	0.400
**Lifestyle** c	**N (%)**				
** Exercises**	75 (38.1)	275 (42.8)	58 (47.2)	241 (42.4)	0.440
** Alcohol**	72 (36.5)	233 (36.3)	50 (40.7)	191 (33.6)	0.457
** Tea**	116 (58.9)	403 (62.8)	80 (65.0)	308 (54.1)	0.011
** Smoking**	108 (54.8)	326 (50.8)	55 (44.7)	273 (48.0)	0.235
**Chronic disease** c					
** Diabetes**	15 (7.6)	70 (10.9)	20 (16.3)	94 (16.5)*	0.002
** Hypertension**	52 (26.4)	212 (33.0)	47 (38.2)	222 (39.0)*	0.007
** Dyslipidemia**	16 (8.1)	54 (8.4)	15 (12.2)	46 (8.1)	0.511
** BPH**	9 (4.6)	42 (6.5)	18 (14.6)*	44 (7.7)	0.006
** PLS**	2 (1.0)	24 (3.7)	7 (5.7)	23 (4.0)	0.133

**Abbreviations:** **ED**, erectile dysfunction; **Mo-Se:** moderate to severe; **BMI**, body mass index; **WC**, waist circumference; **BP**, blood pressure; **HR**, heart rate; **IIEF-5**, 5-item form of International Index of Erectile Function; **FBG**, fast blood glucose; **TT**, total testosterone;**TPSA**, total prostate specific antigen; **FSH**, follicle-stimulating hormone; **LH**, luteotropic hormone; **PRL,** prolactin; **E_2_**, **Estradiol; ALT**, alanine transferase; **TG,** triglyceride; **BPH**, benign prostatic hyperplasia; **PLS**, prostatatis-like symptom; **SD**, standard deviation, **Min**, minimal; **Max**, maximum.

a Data met normal distribution, using One-Way ANOVA analysis;

b Data met non-normal distribution, using K-independent samples analysis;

c Ranked data, using Chi-square tests analysis.

Bonferroni correction *p*: original p×4; *vs. Non-ED, *p*<0.05; ^†^vs. Mo-Se ED, *p*<0.05.

Table 2 showed the associations between sexual status and ED risk factors. Using logistic regression, we found a positive association between moderate to severe ED and old age (odds ratio (OR) = 8.01, 95% CI: 3.62–17.71; *P*<0.001), diabetes (OR = 2.36, 95%CI: 1.16–4.80; *P* = 0.02), hypertension (OR = 1.72, 95%CI: 1.07–2.79; *P* = 0.03), BPH (OR = 3.58, 95%CI:1.55–8.25; *P* = 0.03) and PLS (OR = 5.88, 95%CI: 1.20–28.79; *P* = 0.03); and a positive correlation between asexuality status and old age (OR = 18.49, 95% CI: 10.34–33.05; *P*<0.001), diabetes (OR = 2.40, 95%CI: 1.36–4.25; *P* = 0.003) and hypertension (OR = 1.78; 95%CI: 1.25–2.55; *P* = 0.002).

**Table 2:** Bivariate and multivariate association of impact factors with sexual function.

Predictor	Mo-Se ED Vs. Non-ED				Asexuality status Vs. Non-ED			
			Odds Ratio (95% CI)					
	Unadjusted	*p*	Adjusted	*p*	Unadjusted	*p*	Adjusted	*p*
** Age**								
40–51	1.00		1.00		1.00		1.00	
52–59	2.61(1.16–5.87)	0.02	2.94 (1.26–6.83)	0.01	3.05 (1.71–5.45)	<0.001	2.99 (1.66–5.40)	<0.001
60–64	4.41 (1.97–9.88)	<0.001	3.71 (1.57–8.77)	0.003	8.56 (4.81–15.21)	<0.001	8.26(4.54–15.05)	<0.001
65–80	8.01 (3.62–17.71)	<0.001	7.19(3.03–17.10)	<0.001	18.49(10.34–33.05)	<0.001	17.69(9.59–32.61)	<0.001
** BMI** ≥25	1.57 (0.99–2.51)	0.06	1.55(0.89–2.67)	0.12	1.06 (075–1.49)	0.75	1.04 (0.69–1.57)	0.86
** WC** ≥90	1.34 (0.81–2.21)	0.26	1.49 (0.82–2.73)	0.19	0.75 (0.51–1.11)	0.15	0.81 (0.51–1.29)	0.38
**Lifestyle**								
** Exercise**	0.69(0.44–1.09)	0.11	0.92 (0.55–1.55)	0.76	0.84 (0.60–1.17)	0.29	0.93 (0.63–1.36)	0.71
** Alcohol**	0.84(0.53–1.34)	0.46	0.93 (0.55–1.58)	0.79	1.14 (0.81–1.59)	0.45	1.22 (0.83–1.80)	0.32
** Tea**	0.77 (0.48–1.23)	0.27	0.69 (0.41–1.16)	0.16	1.21 (0.87–1.69)	0.25	0.89 (0.61–1.31)	0.57
** Smoking**	1.50 (0.95–2.36)	0.08	1.29 (0.76–2.18)	0.35	1.32 (0.95–1.82)	0.09	0.95 (0.65–1.39)	0.79
**Chronic disease**								
** Diabetes**	2.36 (1.16–4.80)	0.02	1.76 (0.79–3.93)	0.17	2.40 (1.36–4.25)	0.003	1.79 (0.95–3.36)	0.07
** Hypertension**	1.72 (1.07–2.79)	0.03	1.11 (0.64–1.92)	0.71	1.78(1.25–2.55)	0.002	1.09 (0.73–1.66)	0.65
** Dyslipidemia**	1.57 (0.75–3.31)	0.23	0.79 (0.34–1.85)	0.59	0.99 (0.55–1.80)	0.99	0.65 (0.33–1.28)	0.22
** BPH**	3.58 (1.55–8.25)	0.03	2.08 (0.81–5.29)	0.13	1.75 (0.84–3.66)	0.14	1.15 (0.51–2.61)	0.73
** PLS**	5.88(1.20–28.79)	0.03	4.32 (0.75–25.04)	0.10	4.11 (0.96–17.58)	0.06	3.53 (0.71–17.43)	0.12

**Abbreviations:** **ED**, erectile dysfunction; **CI**, confidence interval; **BMI**, body mass index; **WC**, waist circumference; **BPH**, benign prostatic hyperplasia; **PLS**, prostatatis–like symptom, **Vs.**, versus.

**Multivariate regression:** All variables listed in the table have been included in the multivariate logistic regression model simultaneously.

## Discussion

Our findings, based on nationally representative data from Shanghai, indicated that most middle-aged and old adults had sexual problems, and moreover, a substantial number of men presented asexuality status. Men with asexuality status suffered higher risk factors than moderate to severe ED population and most reasons for their asexuality were associated with erectile dysfunction, while only a few men with asexuality status reported that they had a normal erection during masturbation.

The total prevalence and common risk factors of ED in our study supported the previous research in Asian and Western countries [18], [19], [20], [21]. The established ED risk factors included old age, diabetes, hypertension, BPH and PLS, and old age was the independent risk factor. However, we also found several disparities in this population. The prevalence of ED in men with 40–51 years was 58.6%, which seems to be different from the data in past epidemiological investigations (ranged from 2% to 39% in men between the ages of 40 and 50 years) [22]. The high prevalence of ED in 40–51 years group can be explained as these: firstly, more and more evidence in recent years has shown that the incidence of ED is increasing significantly in young and middle-aged men [23], [24]; secondly, high proportion of mild ED (53.5% in all, not shown in results) presented in this cohort, which is often overlooked in clinical practice [25]; thirdly, Chinese cultural and social influences might result in higher incidence of psychogenic ED presented in middle-aged men [26], while the IIEF-5 scores do not exclude psychological ED [26], [27]. There were no significant associations between ED, dyslipidemia and lifestyles, which might differ from Italy’s research data that patients with dyslipidemia [28] or/and adverse lifestyles [29], [30] were at increased risk of developing ED. These inconsistent findings might originate from the difference of population. Smith et al. [31] found that there was no association between total IIEF-15 score or severity of ED and serum cholesterol and triglyceride levels, and Hall et al [32] also found there was no significant positive association between untreated hyperlipidemia and ED in multivariate model. In our study population, most subjects had older age (64.5 percent >60 years) and suffered increasing systemic diseases, thus their poor health status would urge them to improve their lifestyles (for example, the improvement of diet and physical activity behaviors), which might benefit their control of dyslipidemia and obesity. But on the other hand, these findings suggested that ED in this cohort may be affected more significantly by systemic diseases than by adverse lifestyle factors.

The IIEF (or IIEF-5) scoring system is widely used to evaluate erectile function [33], [34]. However, the questionnaire, taking no account of the men with “no sexual activity in the last 4 weeks”, is limited for evaluating asexuality status which was defined here as having no sexual attempts for more than 6 months. As there are no specific recommendations for evaluating asexuality in the clinical guidelines, the subjects with asexuality status are usually excluded from study populations in most reports. However, the answer to this question is of particular importance as there are a substantial number of people who are at least temporary asexual, specially the old age people [35], [36]. In our study, 37.2% of the middle-age and old men presented asexuality status, thus indicating that the analysis of this subgroup cannot be neglected. In order to clarify the ambiguous status of erectile function (completed ED or normal erectile function) in the cohort with asexuality status, we compared asexuality status with moderate to severe ED and non-ED in terms of risk factors associated with ED. The adjusted hazard ratios of 60–64 years and 65–80 years in respondents with asexuality status were significantly higher than in the cohort with moderate to severe ED by 2.5 fold and 2.2 fold, respectively. Moreover, the risks of diabetes and hypertension in men with asexuality status were higher than in moderate to severe ED men. These findings suggested that the majority of the cases with an asexuality status might be related to a full ED, which is understandable since most men with an asexuality status had lost the ability to a normal sexual intercourse.

In an attempt to verify the foregoing inference, we individually collected the self-report reasons for asexuality in those men without sexual intercourse. The self-report information in our study showed that 52.9% of men in the asexuality status category men regarded “erectile difficulty” as the main reason for this asexuality, which supported the aforementioned verification directly. Furthermore, the complaints of “severe stress”(44.4%), “severe fatigue” (26.3%), “poor sexual relationship” (4.4%) and “low life satisfaction” (3.0%), which represent social, psychological and physical stresses causing adverse effects on sexual activities and erection [37], [38], were also the reasons for the development of asexuality in this population. These findings explained in part why most men with asexuality status suffered erectile difficulty. We noted that most men with an asexual status regarded “do not care about sexuality” (53.5%) and “no longer necessary to have sexuality at this age” (47.7%) as another two main reasons for asexuality, and it seems that men providing these reasons might have a normal erectile function. In fact, the phenomenon involved two aspects: attitudes towards sexuality and lack of sexual interests. As most men with asexual status were married or had a prior active sexual life, the asexuality status here is distinct from permanent asexual condition which is abstention from sexual activity and celibacy resulted from individual’s personal or religious beliefs or/and sexual orientation [39]. Thus the most likely reason for the difference of sexual attitudes might be the concern that sexual activities would do harm to their worsening health with increasing age. The lack of sexual interests might be correlated with the old age (mean 65.70±8.20 years), lower total testosterone (compared with non-ED) and accompanying chronic diseases, which is consistent with data in European Male Ageing Study (EMAS) [40]. A declining serum testosterone levels would lead to a gradual loss of libido [41], and a deficiency of serum testosterone might induce erectile dysfunction by impairing the vasodilation of penile arterioles and cavernous sinusoids [42]. Hence, the different sexual attitudes and low sexual desire were associated with the risk factors of ED as well.

Men with single status and masturbation experience might suggest that they maintained normal erectile function even if they had no sexual intercourse attempts. However, we found that a small proportion of men with asexual status regarded “single, widowed, divorced or separated” (4.3%) as the reasons for asexuality in the study. Although around a quarter of men with asexuality reported they had masturbatory experience, only 35.3% of them (9.5% in all) considered they had normal masturbatory erection. In short, although a variety of reasons for asexuality were reported, most of them could be attributed to erectile difficulty and its risk factors.

This study has several strengths, including a population-based prospective cohort study design, large overall sample size and standardized protocols conducted by trained interviewers. Selection bias was minimized due to the exceptionally high response rates at recruitment (92.5%). Importantly, we defined asexuality status with precision and classified subjects with asexuality as a subgroup for analysis. The exploration of the asexuality status vis-à-vis a life-long asexuality may add to the literature as no specific recommendation for evaluating asexuality exists in clinical practice. However, limitations of this study should be considered for the interpretation of results. Like most similar research studies, one concern is the fact that some data were self-reported, although the interview methods are well accepted as valid. To address this concern, we collected as much objective data as possible to support the self-reported results. Another concern is that we did not collect the pertinent detailed information about the healthy status of the female partners and we did not survey and analyze life-long asexual status which is different from transient asexuality in our population.

In conclusion, the asexuality status was frequent among middle-age to old men, and men with this condition suffered higher risk factors for ED than men with moderate to severe ED. The majority of the asexuality status could be attributed to a condition of full ED, although the reasons for an asexuality status also involved sexual attitudes and interests, sexual partners and masturbation. Further studies are needed to design an appropriate investigation to evaluate the prevalence of organic versus psychogenic erectile function in the population with an asexuality status, and also identify a subsection of permanently asexual men, including much younger men, i.e., down to 18 years old. The latter would also help to define the factors, so far unknown, that may induce in young men a disinterest for an active sexual life.
